# Recent Developments of Tin (II) Sulfide/Carbon Composites for Achieving High-Performance Lithium Ion Batteries: A Critical Review

**DOI:** 10.3390/nano12081246

**Published:** 2022-04-07

**Authors:** Sharif Tasnim Mahmud, Rony Mia, Sakil Mahmud, Sha Sha, Ruquan Zhang, Zhongmin Deng, Meltem Yanilmaz, Lei Luo, Jiadeng Zhu

**Affiliations:** 1 State Key Laboratory of New Textile Materials and Advanced Processing Technology, School of Textile Science and Engineering, Wuhan Textile University, Wuhan 430200, China; tasnimmahmud13047@gmail.com (S.T.M.); shasha@wtu.edu.cn (S.S.); zhangruquan@wtu.edu.cn (R.Z.); zmdeng@wtu.edu.cn (Z.D.); 2 Hubei Key Laboratory of Biomass Fibers and Eco-Dyeing & Finishing, School of Chemistry and Chemical Engineering, Wuhan Textile University, Wuhan 430200, China; mroni_mia@yahoo.com (R.M.); sakilmahmud1105@gmail.com (S.M.); 3 Department of Chemical Engineering, Inha University, Incheon 22212, Korea; 4 Department of Textile Engineering, Istanbul Technical University, Istanbul 34469, Turkey; yanilmaz@itu.edu.tr; 5 Chemical Sciences Division, Oak Ridge National Laboratory, Oak Ridge, TN 37831, USA

**Keywords:** tin (II) sulfide, carbon, lithium ion battery, anode, lithium storage property, high energy density

## Abstract

The ever-increasing worldwide energy demand and the limited resources of fossil have forced the urgent adoption of renewable energy sources. Additionally, concerns over CO_2_ emissions and potential increases in fuel prices have boosted technical efforts to make hybrid and electric vehicles more accessible to the public. Rechargeable batteries are undoubtedly a key player in this regard, especially lithium ion batteries (LIBs), which have high power capacity, a fast charge/discharge rate, and good cycle stability, while their further energy density improvement has been severely limited, because of the relatively low theoretical capacity of the graphite anode material which is mostly used. Among various high-capacity anode candidates, tin (II) sulfide (SnS_2_) has been attracted remarkable attention for high-energy LIBs due to its enormous resource and simplicity of synthesis, in addition to its high theoretical capacity. However, SnS_2_ has poor intrinsic conductivity, a big volume transition, and a low initial Coulombic efficiency, resulting in a short lifespan. SnS_2_/carbon composites have been considered to be a most promising approach to addressing the abovementioned issues. Therefore, this review summarizes the current progress in the synthesis of SnS_2_/carbon anode materials and their Li-ion storage properties, with special attention to the developments in Li-based technology, attributed to its immense current importance and promising prospects. Finally, the existing challenges within this field are presented, and potential opportunities are discussed.

## 1. Introduction

Lithium ion batteries (LIBs), with high energy density, extended cycle life, and environmental friendliness, have been considered to be one of the most appealing energy storage systems, and have played an increasingly significant role in modern civilization [1,2,3,4,5,6]. The progressing advancement of LIBs has brought exceptional enhancements in different parts of their activity [7,8,9,10,11,12], being widely involved on the market of compact electronic devices (e.g., cell phones, workstations, advanced cameras, etc.). Additionally, they have been distinguished as the favored force hotspot for electric vehicles (EVs) and fixed-vitality energy storage. However, state-of-the-art LIBs cannot fulfill the developing need for EVs and huge scope vitality energy storage [13,14,15,16,17], which is mainly caused by the limited capacity (372 mAh/g) of the mostly used graphite anode [13,14,17,18,19,20,21]. Thus, tremendous efforts have been made to fabricate high-capacity anode materials, including elementary substances (i.e., Ge, P, Sb, Si), transition metal oxides (i.e., MnO, V_2_O_5_), metal sulfides (i.e., ZnS, Cu_2_S), etc. [22,23,24,25,26,27,28,29,30,31,32,33,34,35,36,37,38,39,40,41,42]. Among them, SnS_2_ has been attracted remarkable attention because of its low cost, environmental friendliness, and high theoretical specific capacity [43,44,45,46,47]. SnS_2_ has the catenation ability of sulfur and contributes to the enrichment of the chemistry of tin sulfide. It is also possible to include other elements (metal and non-metal) to form trivalent and quadratic tin sulfide structures, as well as ternary and quaternary materials [48,49,50,51,52].

For SnS_2_, tin particles can exist in various oxidation states and different coordination structures, and sulfur ions have an enormous electronegativity and solid polarizability [16,53,54,55,56,57]. Li-ions diffuse from the octahedral gap position, where the energy is most supported, to the adjacent octahedral gap position, through the tetrahedral gap position. The expansion of the layer spacing is beneficial for reducing the diffusion barrier [58,59]. In addition, the energy density of SnS_2_-based LIBs can reach as high as 286 Wh/kg, which is much better than those of commercial graphite [60]. Despite these advantages, SnS_2_ has certain drawbacks that hinder its broad application, such as the non-negligible volume change issue and the comparatively poor initial Coulombic efficiency (CE), which is related to the irreversible synthesis of Li_2_S and Li_x_SnS_2_ [61,62,63].

Overall, the commercial applications of SnS_2_-based anode materials are currently limited in large part by the following issues: (1) significant initial irreversible capacity loss as a result of the creation of thick solid electrolyte interphase (SEI) throughout the cycling process; (2) the large volume change that occurs during the charge/discharge process, which results in electrode pulverization and the loss of electrical contact with the current collector, leading to fast capacity fading along with poor cycling performance [64,65,66,67]. Many efforts have recently been concentrated on addressing the aforementioned difficulties, as well as advancing the use of SnS_2_-based anode materials in LIBs for practical applications. It has been found that hybridizing SnS_2_ with other materials—including nanocarbon [68,69], graphene [70,71], and MXene [72]—or doping SnS_2_ with other additives—such as Co [39], Ce [73], Mo [74], etc.—can significantly improve the overall conductivity and structural resilience of SnS_2_-based electrodes, thus resolving the issues mentioned above. As most commonly modified materials, carbon materials (i.e., amorphous carbon, carbon nanotubes, graphene, etc.) have been extensively explored because they can improve the electrical conductivity of the electrode, and can reduce the particle agglomeration of active materials, enhancing the utilization of active materials and extending their lifespan [75,76,77,78].

This paper has reviewed the most recent developments in SnS_2_/carbon anodes for LIBs. The structural properties of different composites using SnS_2_ clearly demonstrate the importance of preparation process. To fully use all the potential advantages of SnS_2_ in LIBs, endeavors have been made to handle the previously mentioned issues and push SnS_2_-based anode materials to handy applications. The morphological design and fabrication of electrode materials tremendously affect the electrochemical performance of LIBs; thus, the large-scale study of those material-based anodes is essential. This review highlights the most recent developments with thorough discussion in the microstructure, morphology, rational synthesis, and electrochemical performance of SnS_2_-based anode materials in LIBs with a goal to provide more insights in this area. The future challenges and research directions for practical, advanced SnS_2_-based anodes are also proposed at the end.

## 2. Working Mechanisms of SnS_2_-Based Anodes in LIBs

During the past decades, Sn-based materials, particularly SnS_2_, have played a major role in LIBs due to their layered structure, which allows them to provide optimum space for Li-ion intercalation [79,80,81,82,83]. It has also been demonstrated that the advantage of having layers in the structure of the crystal can be used to accommodate Li-ions [84]. The neighboring sulfur layers in SnS_2_ are held together by weak van der Waals contacts [85]. The lithiation procedure of SnS_2_ can be separated into two phases. When the Li content (x in Li_x_SnS_2_) is under 1, the volume extension is not remarkable, and only the S particles trap electrons from Li-ions. When the Li content is more than 1, the Sn^4+^ cations are fundamentally decreased, the 3 S-Sn-S layers deteriorate bit by bit, and an Li_x_S_2_ (1 ≤ x ≤ 3) layer is framed between the 2 Sn monolayers, and the volume expansion of SnS_2_ subtly reduces the intensity of Li 2p states. The anode’s stability may be jeopardized due to the lithiation-induced volume expansion and crystal structural change. During lithiation/delithiation, Sn-based anode materials always experience 200–300% volume expansion [61,62,63].

It has been proposed that the electrochemical reaction mechanisms of SnS_2_ with Li-ions are presented in Equations (1) and (2) [86]:

SnS_2_ + 4Li^+^ + 4e^−^ → Sn + 2Li_2_S, (1)

Sn + xLi^+^ + xe^−^ ↔ Li_x_Sn (0 ≤ x ≤ 4.4), (2)

The high theoretical capacity of 645 mAh/g is derived from the reversible reaction (1) of Sn in SnS_2_ with 4.4 mols of lithium. Despite this, during the conversion reaction of lithiated SnS_2_, 4 mols of lithium are consumed in the irreversible formation of Li_2_S. As a result, if the irreversible reaction is made reversible, then the theoretical capacity of SnS_2_ could be as high as 1231 mAh/g (8.4 mol Li^+^ per mol SnS_2_) [87]. Lithiation causes SnS_2_ to decompose into metallic tin and Li_2_S during the first discharge. Tin alloys/dealloys up to the theoretical limit of Li_4_._4_Sn, and Li_2_S act as an inert matrix that surrounds the active Sn grains during substantial charge and discharge processes [86]. Li-ions can intercalate to some extent into the SnS_2_ layers without generating phase dissolution, according to earlier publications [45,50]; hence, the reaction can be separated into three phases, as follows in Equations (3)–(5):

SnS_2_ + xLi^+^ + xe^−^ → Li_x_SnS_2_, (3)

Li_x_SnS_2_ + (y − x)Li^+^ + (y − x)e^−^ → Li_y_SnS_2_, (4)

Li_y_SnS_2_ + (4 − y)Li^+^ + (4 − y)e^−^ → Sn + 2Li_2_S (0 < x < y ≤ 2), (5)

To enhance the energy density and capacity of battery materials, a detailed understanding of electrode thermodynamics and chemistry is required [83,84]. First principles have been utilized to study the Li-ion intercalation and diffusion in pristine and modified SnS_2_ interlayers. The data suggest that Li intercalation prefers the octahedral interstitial location. The minimum energy path of Li-ion diffusion in the SnS_2_ interlayer is explored. Researchers have discovered that Li atoms spread from one energetically favorable octahedral interstitial location to the next [63]. The results of this study suggest that regulating the inactive morphologies of SnS_2_-based anode materials may be a viable strategy for improving their electrochemical performances.

## 3. Pure SnS_2_

The SnS_2_ anode material for LIBs was first reported in 1998 by T. Brousse and his team [86]. SnS_2_ is an n-type semiconductor “layered compound”, with a hexagonal cadmium iodide (CdI_2_) structure that has the potential to own a high capacity [88,89,90,91,92]. SnS_2_ can host molecular guest species in vacancies between its neighboring sulfur layers because of its layered structure, similarly to how Li-ion is embedded in graphite [50,66,83,84,93]. For pure SnS_2_ anodes, different structures have been designed and investigated, such as nanoparticles [67,83], nanosheets [50,94,95,96,97], nanowalls [98,99], and nanoflowers [100,101,102,103], that show different capacities according to their morphologies.

For example, Momma et al. [92] observed that the amorphous SnS_2_ powder could be a viable candidate material for LIBs. An aqueous solution of SnCl_4_ and thioacetamide was sonicated in air at ambient temperature for 30 min to improve crystallinity before being annealed at 400 °C. The cell with the unannealed SnS_2_ electrode had an initial capacity of 300 mAh/g at a current density of 50 mA/g. After annealing, the capacity of SnS_2_ increased to above 600 mAh/g. The results showed that the crystalline morphology of annealed SnS_2_ has been revealed as a possible anode candidate for LIBs because it could accelerate the lithiation process.

Reducing the particle size of Sn-based materials to the nanoscale range is an efficient technique to improve cycling stability. Various methods have been developed for the synthesis of SnS_2_ nanostructures with various diameters (from 10 to 100 nm) and morphologies (i.e., nanoparticles, nanorods, nanobelts, nanotubes, and nanosheets) [104,105,106,107,108,109,110,111,112,113,114,115,116]. Kim et al. synthesized novel crystalline SnS_2_ nanosheets/nanoplates and applied them as anode materials for LIBs [50]. SnS_2_ nanosheets from ~1.6 to ~26 nm were successfully prepared via a simple, catalyst-free solvothermal route, without surfactants/functional groups. Ethylene glycol was used as a reducing agent by capping the Sn-ion source, resulting in creating a polymer network and the prevention of nanosheet aggregation. Li-ions could be embedded in the SnS_2_ layer to some extent without causing phase decomposition for nanosheet structures.

**Figure 1:**
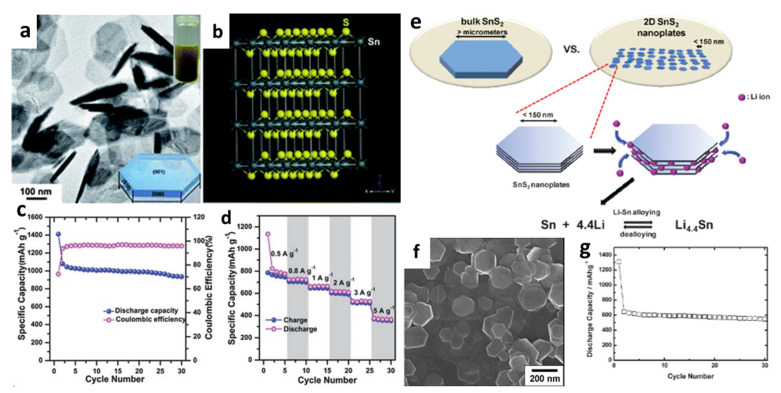
(**a**) A TEM image of hexagonal SnS_2_ nanoplates. Inset: (top) photograph of SnS_2_ nanoplate solution; (bottom) schematic diagram of a SnS_2_ nanoplate. (**b**) The supercell structure of a SnS_2_ crystal with A = 3a, B = 3b, C = 3c. (**c**) Cycling performance of the SnS_2_ nanoplate electrode at a current density of 0.2 A/g and (**d**) charge-discharge capacities at various current densities from 0.5 to 5 A/g. Reprinted with permission from Ref. [97]. Copyright 2013 RSC. (**e**) A schematic of lithiation processes for bulk versus nanoplates. (**f**) An SEM image of SnS_2_ nanoplates; (**g**) life cycle performance of the SnS_2_ electrode. Reprinted with permission from Ref. [94]. Copyright 2008 John Wiley and Sons, Inc.

**Figure 2:**
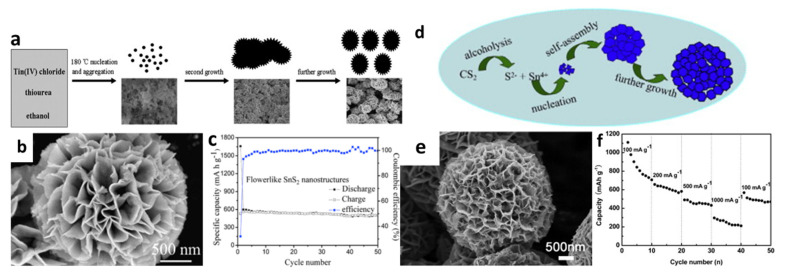
(**a**) Schematic illustration of the morphological evolution process, (**b**) an SEM image, and (**c**) cycling performance of the 3D flower-like SnS_2_. Reprinted with permission from Ref. [66]. Copyright 2010 Elsevier. (**d**) Schematic illustration of the morphological formation process, (**e**) an SEM image, and (**f**) rate capabilities of SnS_2_ hierarchitectures. Reprinted with permission from Ref. [102]. Copyright 2013 Elsevier.

During the lithiation/delithiation process, many efforts have been undertaken to minimize the volume change and improve cycle performance. Du et al. [97] introduced an *eco*-accommodating and conservative manufacturing method for two-dimensional (2D) layered SnS_2_ nanoplates by one-pot synthesis using SnCl_2_·2H_2_O powder (Figure 1a). Figure 2b shows the crystal structure of SnS_2_ nanoplates with alternating S-Sn-S layers and S-S layers along the z-axis (c-axis). The final fabricated cell exhibited highly reversible capacity and good capacity retention after 30 cycles (Figure 1c,d). Seo et al. [94] discovered 2D layered nanostructures by thermal decomposition and provided better cyclability due to their unique nanoscale phenomena below 150 nm (Figure 1e,f). The determined average discharge capacity could be up to 583 mAh/g, which was 90% of the maximum theoretical reversible value and 1.6 times the commercial carbon electrode (372 mAh/g), as shown in Figure 1g. This exhibited greatly improved host capabilities as an active LIB electrode because of its unique shape, which consists of a finite, lateral sized, and well-defined layered structure.

The vertically aligned 2D SnS_2_ nanowalls can also serve as an ideal anode material for LIBs. Liu et al. [98] performed a simple chemical bath deposition method to prepare SnS_2_ nanowall arrays grown directly on copper foils. The shape of these arrays offers numerous benefits for enhancing electrocatalytic activity. Because there are more space between neighboring nanowalls, the electrolyte may readily diffuse into the inner area of the electrode, and the volume change associated with Li^+^ insertion and extraction can be maintained. In the meantime, a simple, biomolecule-assisted technique was used to produce vertically aligned SnS_2_ ultrathin nanosheet arrays on Sn substrate by Zhong et al. [99]. A facile, L-cysteine-assisted hydrothermal strategy was devised to manufacture a graphene-like SnS_2_ film comprising 2–5 atomic layers on Sn foils. The electrochemical discharge capability of the cell with ultrathin SnS_2_ nanosheets was 690 mAh/g at 3C that was near to the theoretical limit.

The formation of 3D flower-like structures was initially proposed early in 2010 by Liu et al. [66]. The electrochemical characteristics of flower-like SnS_2_ systems were remarkable according to the achieved results. These flower-like SnS_2_ structures were prepared by a solvothermal ethanol method and produced nanoplates with thicknesses of about 5–10 nm, which revealed a reversible capacity of about 502 mAh/g after 50 cycles at a current density of 200 mA/g (Figure 2a–c). Wu et al. [102] used CS_2_ for the dissolve in ethanol to form a homogeneous solution, in which CS_2_ acted as a sulfur donor throughout the solvothermal process and S^2-^ was released as a source of sulfides (Figure 2d,e). The cell with such prepared electrodes could have capacities of 706.7, 582.4, 432.8, and 210.8 mAh/g, at current densities of 100, 200, 500, and 1000 mA/g, respectively, and it was reversible back to 471 mAh/g when lowering the current density to 100 mA/g (Figure 2f). The overall capacity retained was 73% of the theoretical reversible capacity. The enhanced performance might be because the diffusion distance for ionic and electronic transport was greatly reduced, caused by the specific porous structures of the thin nanosheets, which were accessible for electrolytes and sufficiently dissipated the mechanical stress resulting from the severe volume change during Li-ion uptake/removal. Compared with SnS_2_ nanoparticle anodes, the layered porous structure and flower-like building blocks of these SnS_2_ nanoflowers make the redox reaction and charge transfer kinetics at the electrode faster, and show a much higher discharge capacity than SnS_2_ nanoparticles [116].

Among those morphologies, nanowall- and nanoflower-based SnS_2_ anodes provide more stable cyclic ability. However, within similar morphology, the particle or plate size has a significant impact on the lithiation and delithiation process, depending on the method, reaction time, reaction temperature, annealing, and crystallinity of the material. The electrochemical performance of pure SnS_2_-based anode is summarized in Table 1. For tin-based electrode materials, an increase in current density usually results in significant capacity fading. As can be seen, bare SnS_2_-based anode materials continue to be obviously harmed by large initial irreversible capacity losses, severe internal stress, and loss of electrical contact with the current collector. Some primary strategies have been adopted to address these current issues. The initial objective is to create a variety of novel, nanostructured SnS_2_ materials, including nanoparticles, nanosheets, and nanoflowers. Not only may nanoscale materials reduce the diffusion length of electrons and lithium ions, but they can also mitigate the large volume impact. However, the nanostructured or porous-structured material alone does not appear to be capable of completely resolving the abovementioned issues, particularly at long cycles and high rates. Thus, using cost-effective carbonaceous materials along with their easy processes is a unique technique which has been considered for increasing the capacity and cycling stability of SnS_2_.

## 4. SnS_2_/Carbon Composites

### 4.1. Amorphous Carbon/SnS_2_ Composites

Amorphous carbon has garnered considerable interest in energy-storage applications due to its high electrochemical activity and inexpensive cost [117,118,119,120,121,122,123,124,125]. It has been widely shown to improve the capacity and cycling stability of electrodes.

For instance, Kim et al. [121] first discovered carbon-coated SnS_2_ nanoparticles, in a study in which SnS_2_ powder was extracted from SnCl_4_·5H_2_O and thioacetamide by the solvothermal method and the carbon coating was derived from glucose. After 50 cycles, the C-SnS_2_ nanocomposite had a high reversible capacity of 668 mAh/g at a current density of 50 mA/g, superior to bare SnS_2_ nanoparticles in terms of cycle performance and rate capability, which owed to the conductive carbon shells and their close association with inert nanoscale SnS_2_ materials. Furthermore, a simple, high-energy ball-milling method was developed to synthesize SnS_2_/carbon (SnS_2_/C-x, x = 40, 50, 60 wt.%) nanocomposites in order to study the effect of carbon contents on the overall performance by Zhao et al. [122]. The results indicated that the SnS_2_/C-50 nanocomposite exhibited a remarkably high capacity of 700 mAh/g and stable cycle capacity of 540 mAh/g after 100 cycles at the same current rate of 100 mA/g. SnS_2_ NPs were uniformly implanted inside the graphite nanoparticles network after the ball-milling method, which could provide a large number of Li-ion storage sites, excellent electronic conductivity, and rapid ion diffusion, as well as a reduction in SnS_2_ volume expansion during cycling. Li et al. [123] prepared a 3D mesoporous carbon anchored with SnS_2_ nanosheets (MC-SnS_2_ NSs) by sonochemical reflux method with the structural features of both the 2D nanosheet and the 3D porous carbon matrix, which were expected to show improved Li storage efficiency. The composite showed better cyclic performance and improved structural stability compared with the bare-nanoplate-based SnS_2_-C anode. A stable discharge of 428.8 mAh/g at 100 mA/g after 50 cycles with a retention of 64.4% could be achieved by the MC-SnS_2_ NSs.

**Figure 3:**
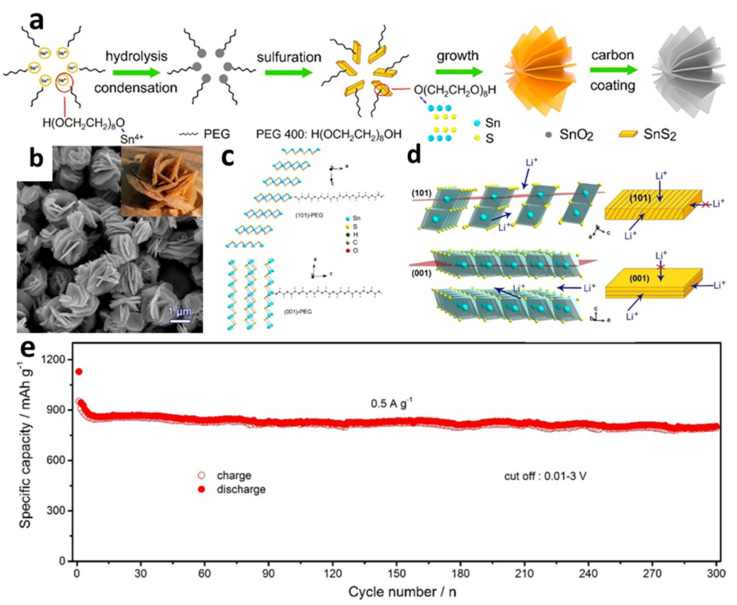
(**a**) Schematic illustration of the formation process; (**b**) SEM images of carbon-encapsulated SnS_2_ nanoplates; (**c**) structural models of terminated (101) and (001) surfaces of SnS_2_ with adsorbed PEG for first-principles calculation; (**d**) schematic illustration of Li-ion insertion; (**e**) long-term cycling performance of carbon-encapsulated SnS_2_ nanoplates with the (101)-oriented plane. Reprinted with permission from Ref. [56]. Copyright 2017 ACS.

Compared with other regularly utilized carbon sources, different biomass-derived carbons have also been employed with the benefits of low cost and environmental friendliness [124]. An innovative SnS_2_/biochars (SnS_2_/B) composite with a hierarchical structure composed of SnS_2_ nanosheet arrays and biochars carbonized from chewed sugarcane was effectively generated using a simple one-step hydrothermal method [125]. The constructed cell with SnS_2_/B could deliver a high initial discharge specific capacity of 1107.4 mAh/g at 100 mA/g with a CE of 54.8%. Zhang et al. [56] fabricated carbon-encapsulated flower-like SnS_2_ nanoplates with (101) plane orientation by a hydrothermal method, with polyethylene glycol (PEG 400) as a surfactant (Figure 3a–c). The SnS_2_ nanoplates synthesized without PEG mainly grew along the (001) plane. The cell with the prepared material showed an excellent capability of 796 mAh/g at a current density of up to 2 A/g along with exceptional cycle stability. The cycle attenuation rate of the cell tested at 0.5 A/g for 300 cycles was only 0.05% (Figure 3e). The outstanding results might be ascribed to the use of highly (101) faceted preferred orientation in the design of the microstructures, creating a quick and long-lasting highway for Li-ion diffusion, resulting in rapid reaction kinetics (Figure 3d). Using a hydrothermal synthesis process coupled with membrane technology, flower-like SnS_2_ nanosheets, evenly fixed in the pores of the carbon membrane (SnS_2_-CM), were produced by Liu et al. [69]. The unique design proved that membrane technology supplied an abundant membrane pore space for uniform SnS_2_ nanosheet development via a C-S covalent connection. For LIBs at 50 mA/g, the highest reversible capacitance could be up to 808.9 mAh/g, which was because thin SnS_2_ nanosheets emerged in the membrane hole and surface, enabling the SnS_2_ cm a 3D interpenetrating network of porous morphology. The novel 3D porous structure not only assisted fast ion transit channels and lowered diffusion length, but also provided ample void space for SnS_2_ nanosheet volume growth during long-term cycles. The C-S covalent bond also maintained a close contact between SnS_2_ and the carbon membrane, contributing to structural stability.

However, SnS_2_ with amorphous carbon typically has a low reversible capacity. Besides, a better rate capabilty is desirable for next-generation LIBs. Thus, alternative carbon-based materials, such as carbon nanotubes (CNTs) and graphene, are being investigated in combination with SnS_2_ in order to increase their specific capacity and rate capability, and also resolve the issues related to pure SnS_2_-based anodes.

### 4.2. CNTs/SnS_2_ Composites

SnS_2_ combined with CNTs is another way to overcome the shortcomings associated with bare SnS_2_ anodes. CNT-based materials may be beneficial for charge transfer and electrode stability, improving the electrochemical performance [126,127,128,129,130,131,132,133,134,135].

**Figure 4:**
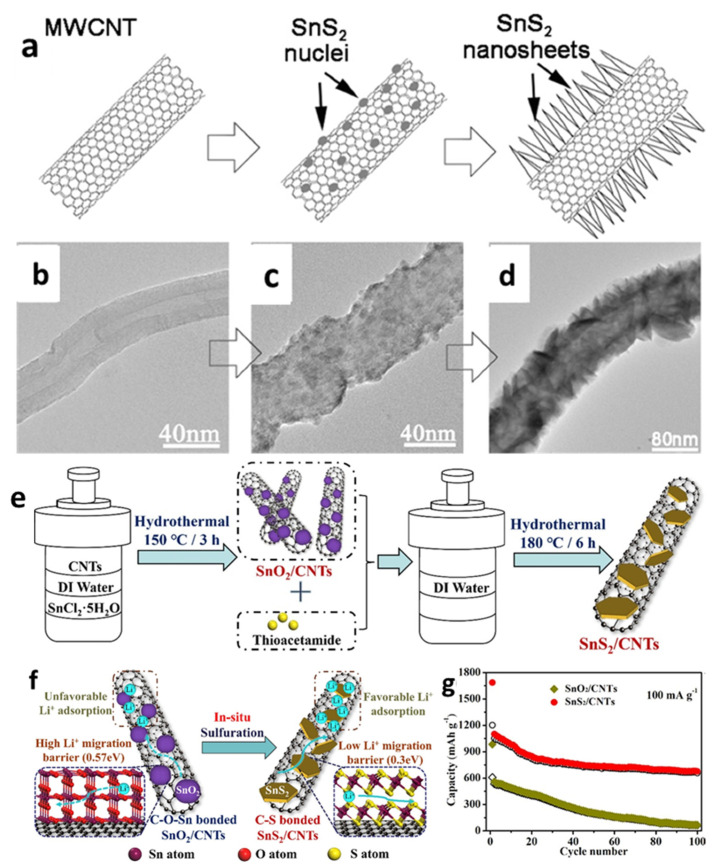
(**a**) Schematic illustration for the growth process of the SnS_2_ NS@MWCNTs and (**b**–**d**) their TEM images. Reprinted with permission from Ref. [126]. Copyright 2011 ACS. (**e**) The synthesis procedures diagram; (**f**) illustration of the Li storage advantage; (**g**) cycling performance of the SnS_2_/CNTs composite. Reprinted with permission from Ref. [48]. Copyright 2021 Elsevier.

Zhai et al. [126] first reported SnS_2_ nanosheets on multiwall CNTs (MWCNTs) by chemical vapor deposition with a tube diameter around 80–90 nm. SnS_2_ nanosheets and nanoflakes were uniformly anchored on CNTs to form SnS_2_/CNT composite anodes with SnS_2_ sheaths of different thicknesses, which exhibited higher Li storage capacity and better cycle performance compared with pure SnS_2_ (Figure 4a–d). Sun et al. [133] also synthesized SnS_2_ nanoflakes decorated on a MWCNT structure through a simple solution–phase method. The cell with the SnS_2_/MWCNTs composite demonstrated initial discharge and charge capacities of 1416 and 518 mAh/g, respectively, and could maintain a reversible capacity of 510 mAh/g after 50 cycles at a current density of 100 mA/g. The improved performance might be attributed to the morphological properties of SnS_2_ flakes and the inclusion of MWCNT, that could reduce volume change throughout the cycle, offer more active sites to accept Li^+^, and accelerate the conductivity of the active material. Differently from the above fabricating processes, a SnS_2_/CNTs composite was also produced via a hydrothermal process by in situ vulcanization of SnO_2_/CNTs by Cheng et al. [48]. In the prefabricated SnO_2_/CNTs composite, SnO_2_ nanoparticles with diameters less than 5 nm were completely coated on the CNTs via Sn-O-C bonding. SnO_2_ nanoparticles were converted into SnS_2_ hexagonal nanosheets during the in situ sulphuration reaction, and the Sn-O-C bonding was replaced by C-S bonding. The cell with the obtained SnS_2_/CNTs exhibited superior electrochemical performance, which could deliver an initial reversible capacity of 1202 mAh/g and a capacity of around 660 mAh/g after 100 cycles at 100 mA/g (Figure 4e–g).

**Figure 5:**
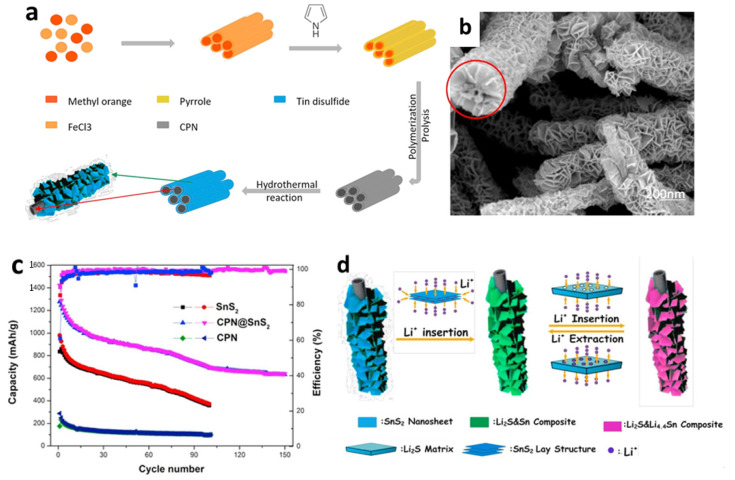
(**a**) Formation mechanism, (**b**) an SEM image, (**c**) cycling performance, and (**d**) a schematic illustration of the Li insertion/extraction mechanism of CPN@SnS_2_ composites. Reprinted with permission from Ref. [127]. Copyright 2017 Elsevier.

In addition, polypyrrole, which is a one kind of carbonaceous substance, is a prospective additive for improving the electrochemical performances of LIBs due to its ease of synthesis, low cost, strong electron conductivity, and environmental stability. Polypyrrole works as a matrix to support the internal stress of electrodes that experience extreme volume changes, as well as providing a conducting backbone for the active materials [135]. Chen et al. [127] prepared composites with a higher initial CE by combining polymerization and hydrothermal process (Figure 5a,b). Two-dimensional SnS_2_ nanosheets were used to adorn carbonaceous polypyrrole nanotubes with the interweaving twisted SnS_2_ nanosheets, reducing volume change during electrochemical cycling and providing more active sites to react with Li-ions. Figure 5c shows that the initial discharge capacity of carbon polypyrrole nanotubes (CPN)-coated SnS_2_ nanosheets was 1422 mAh/g at a current density of 60 mA/g, with a reversible capacity of 699.2 mAh/g after 100 cycles. The excellent electrochemical performance of CPN@SnS_2_ composite anode material derived from a unique structure was due to the insertion of conductive CPN, that substantially enhanced the electronic conductivity of the whole anode, allowing for fast electron transmission, as depicted in Figure 5d.

The electrochemical performance of SnS_2_-CNT-based anodes has been widely explored, and their corresponding results are summarized in Table 2. In order to improve the conductivity of SnS_2_-based materials, it is believed that the combination of electronically conductive agents, such as CNTs, is an effective strategy. An additional effective route is through the morphology-controlled SnS_2_-CNT synthesis of nanostructured, active materials, such as nanowire, nanotubes, nanoflakes, and nanosheets. These nanostructures can shorten the pathway lengths of Li^+^ and compensate for volume change due to their large surface-to-volume ratio, which makes them ideal for use in LIBs. Additionally, SnS_2_-graphene-based composites are currently used for further improving electrochemical performance because they allow enormous concentrations of Li^+^ to adsorb and desorb during charging and discharging cycles.

### 4.3. Graphene/SnS_2_ Composites

Graphene is a novel, 2D, “aromatic” single molecule with high electron mobility, a unique electrical structure, high thermal conductivity, mechanical strength, and a large surface area, which has attracted unprecedented attention [136,137,138,139,140,141,142,143,144,145,146,147,148,149,150,151,152,153,154,155,156,157,158,159,160,161,162,163,164,165,166,167]. Many studies have been conducted to design novel SnS_2_/graphene anode materials for LIBs with different nanostructures to improve the electrochemical properties, including nanoparticles/nanocrystals [53,54,137,142,156,158,161,163], nanosheets/nanoplates [141,143,144,150,151,152,153], and nanoflowers [49].

**Figure 6:**
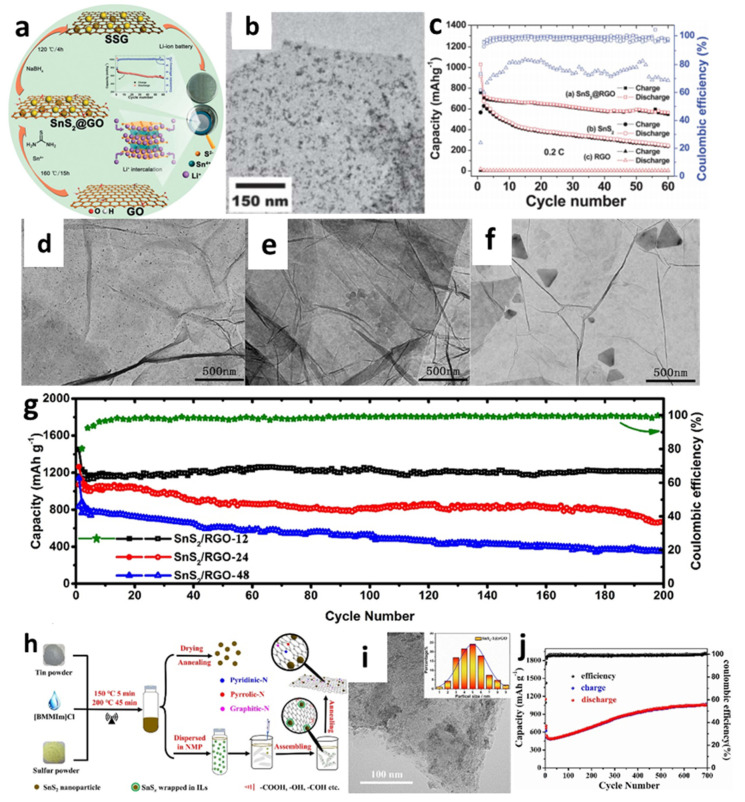
(**a**) Synthetic process, (**b**) a TEM image, (**c**) cycling performance of SnS_2_@RGO composite. Reprinted with permission from Ref. [138]. Copyright 2012 RSC. TEM images of (**d**) SnS_2_/RGO-12, (**e**) SnS_2_/RGO-24, and (**f**) SnS_2_/RGO-48; (**g**) cycling performance of SnS_2_/RGO. Reprinted with permission from Ref. [161]. Copyright 2020 Elsevier. (**h**) Schematic illustration of the synthesis, (**i**) a TEM image, and (**j**) cycling performance at a current density of 500 mA/g of SnS_2_@RGO. Reprinted with permission from Ref. [163]. Copyright 2021 Elsevier.

For instance, Yin et al. [138] decorated SnS_2_ nanocrystals on a reduced graphene oxide (RGO) sheet through the combination of hydrothermal and reduction methods (Figure 6a,b). The cell with the obtained composites showed better cyclic performance with a reversiable capacity of 820 mAh/g at a current rate of 0.2 C after 30 cycles compared with a pure SnS_2_ anode (Figure 6a–c). Controlling the particle size of electrode materials has been acknowledged as an effective approach for improving the cycle stability and rate characteristics of LIBs [67]. Thus, a simple, one-step hydrothermal process for fabricating composites containing size-tunable tin disulfide on SnS_2_-RGO (Figure 6d–f) was investigated by Zhao et al. to thoroughly explore the effect of particle size on the electrochemical properties of the material [161]. To demonstrate the morphological, size-dependent properties, the particle sizes of SnS_2_ nanoparticles were changed by varying the length of the hydrothermal process with three different heat-treatment times (12, 24, or 48 h). The collected samples were marked as SnS_2/_RGO-12, SnS_2_/RGO-24, and SnS_2_/RGO-48, respectively. After 12 h of hydrothermal treatment, the ultrafine SnS_2_ particles (12 nm) were evenly spread over the graphene nanosheets. It is seen from Figure 6g that, after 200 cycles at 0.1 A/g, a high reversible capacity of 1211 mAh/g remained, which was because the prepared samples had more active sites and increased transport kinetics, thus yielding significant enhancement in electrochemical performance. Mei et al. [156] reported ultrasmall SnS_2_ nanocrystals decorated on flexible RGO through a refluxing method. The supplied composite with a high surface-to-volume ratio could enhance Li atom absorption on both sides of the sheet and porous architectures, enabling the RGO nanosheet to offer enough room for Li^+^ storage. The cell with such materials exhibited good capacity retention even at high rates of 1 C and 5 C with the capacities of 773 mA h/g and 415 mAh/g, respectively, after 450 cycles, which were significantly better than the previous hydrothermal-based studies. SnS_2_@RGO nanocomposites were also created using a novel ionic-liquid-assisted method, which employed SnS_x_ precursors by reacting elemental tin and sulfur in the ionic liquid, 1-butyl-2, 3-dimethylimidazolium chloride (Figure 6h,i) [163]. Exceptionally high reversible capacity and cycle stability could be achived by using the obtained composite. A discharge-specific capacity reached 1045.8 mAh/g, even after 700 cycles at a current density of 500 mA/g, as shown in Figure 6j. The improved reversible capacity of the SnS_2_@RGO electrode was explained by electrolyte breakdown at the low potential to create an organic polymeric/gel-like layer due to the “pseudo-capacitance-type behavior” that activated the active material under deep cycling.

**Figure 7:**
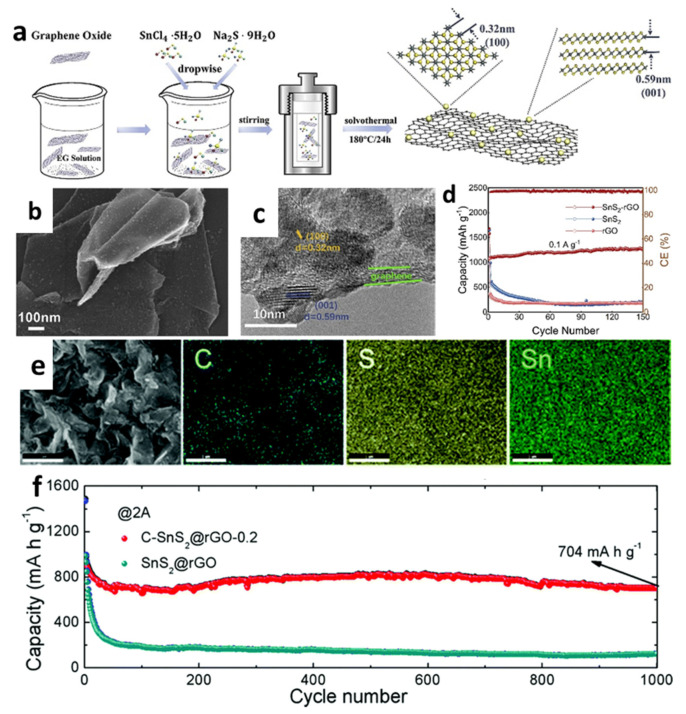
(**a**) Synthesis procedures diagram, (**b**) TEM and (**c**) HRTEM images, and (**d**) cycling performance of SnS_2_@GNS. Reprinted with permission from Ref. [162]. Copyright 2020 Elsevier. (**e**) An SEM image and EDX mapping of C, S, and Sn elements; (**f**) long-term cycle stability of C-SnS_2_@RGO. Reprinted with permission from Ref. [166]. Copyright 2020 RSC.

Furthermore, a homogeneous layer of SnS_2_ nanoparticles was grown on graphene nanosheets (SnS_2_@GNS) and linked by covalent bonds using the solvothermal method (Figure 7a–c) [162]. The I_D_/I_G_ values of SnS_2_@GNS and GNS were calculated to be 1.44 and 1.22, respectively, showing that SnS_2_@GNS had more flaws. High-level flaws in graphene can accelerate ion and electron migration, improve electrochemical reaction kinetics, and offer more active sites for Li-ion adsorption and intercalation [146]. As displayed in Figure 7d, the cell with SnS_2_@GNS delivered a capacity of 1250.8 mAh/g after 150 cycles at 0.1 A/g. In addition, Li et al. [166] prepared SnS_2_ nanocrystals (NCs) through the one-pot solvothermal method using carbon shells attached to RGO by C-S covalent bonding (Figure 7e). The well-controlled carbon shells offered long-term protection for SnS_2_ NCs against electrolyte corrosion and structural pulverization. Carbon shells could act as mediums, enhancing C-structural SnS_2_@RGO’s stability and conductivity. It is demonstrated that LIBs had superior rate capabilities and cycling stability (capacity retention of 74.7% after 1000 cycles at 2.0 A/g, as shown in Figure 7f).

**Figure 8:**
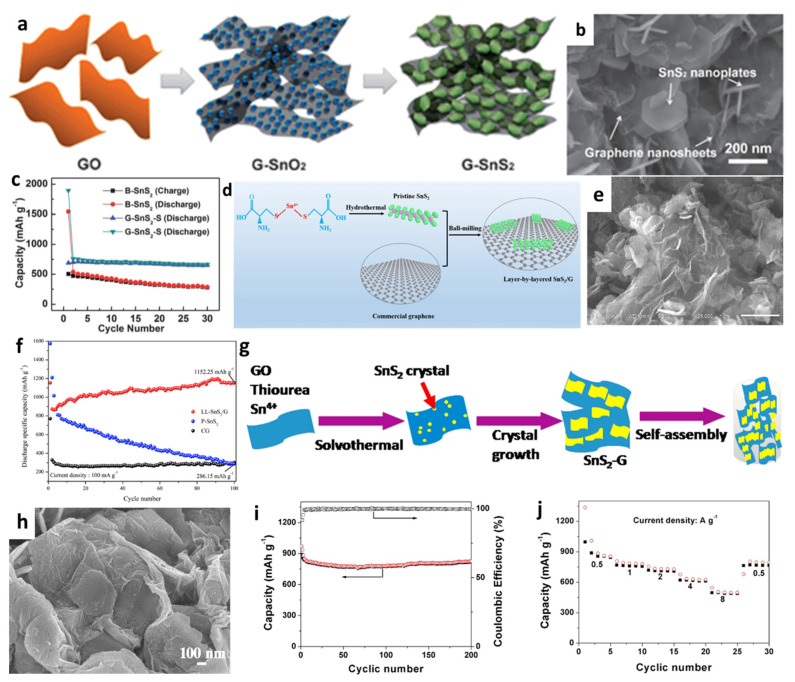
(**a**) Illustration of the formation, (**b**) an SEM image, and (**c**) cycling performance of the G–SnS_2_. Reprinted with permission from Ref. [142]. Copyright 2012 RSC. (**d**) Schematic illustration of the formation, (**e**) an SEM image, and (**f**) cycling performance of LL-SnS_2_/G. Reprinted with permission from Ref. [146]. Copyright 2018 Elsevier. (**g**) Schematic formation, (**h**) an SEM image, (**i**) cycling performance, and (**j**) rate capability of SnS_2_-G. Reprinted with permission from Ref. [151]. Copyright 2016 Elsevier.

Moreover, Luo et al. [142] developed a new porous nanostructure composed of 2D graphene–SnS_2_ (G-SnS_2_) by transforming SnO_2_ nanoparticles into 2D SnS_2_ nanoplates directly on/between graphene nanosheets via a solution approach followed with a chemical vapor deposition (CVD) process (Figure 8a,b). The cycling performace in Figure 8c showed that the cell with the prepared G-SnS_2_ had a stable capacity of 650 mAh/g after 30 cycles at 50 mA/g, while the reversible capacity of bare SnS_2_ gradually decreased to 277 mAh/g. Xia et al. [146] synthesized pristine SnS_2_ nanosheets with a thickness of 5 nm by a hydrothermal process, and then uniformly layered SnS_2_ on graphene sheets to produce layer-by-layer nanosheets (LL-SnS_2_/G) through the ball-milling method (Figure 8d,e). When used as anodes for LIBs, the capacity reached 1152.25 mAh/g after 100 cycles at a current rate of 100 mA/g, as shown in Figure 8f. The excellent electrochemical performance was attributed to the synergistic effect between SnS_2_ nanoplates with high specific capacity and conductivity of graphene, which buffered the volume change and provided an effective physical barrier between the active materials and the electrolyte to suppress the shuttle effect of polysulfides formed during delithiation processes. Chen et al. [157] used reflux condensation and hydrothermal methods to grow SnS_2_ nanoplates on the surface of RGO nanosheets. When the GO concentration was 15%, the SnS_2_/RGO electrode exhibited the excellent electrochemical performance, which showed capacities of 776, 715, 635.6, 595.2, 517.5, and 447.1 mAh/g at current densities of 0.2, 0.5, 1, 2, 5, and 8 C, respectively. In addition, 3D nanoplate-based SnS_2_/graphene was synthesized through a facial solvothermal method by Zhang et al. [151], in which SnS_2_ nanoplates with an average thickness of 3.6 nm were well dispersed and tightly contacted onto graphene substrates (Figure 8g,h). The cell with SnS_2_-G achieved a very stable capacity of 826 mAh/g over 200 cycles at 500 mA/g. The specific capacities of 854, 780, 728, 625, and 498 mAh/g were obtained under the conditions of 0.5, 1, 2, 4, and 8 A/g, respectively (Figure 8i,j). The enhanced electrochemical performance of the cell was because of the enormous surface area of 2D hybrid materials, the highly conductive and flexible graphene matrix, the 3D design, the facilitated electrolyte filtration, and the smooth ion transport.

**Figure 9:**
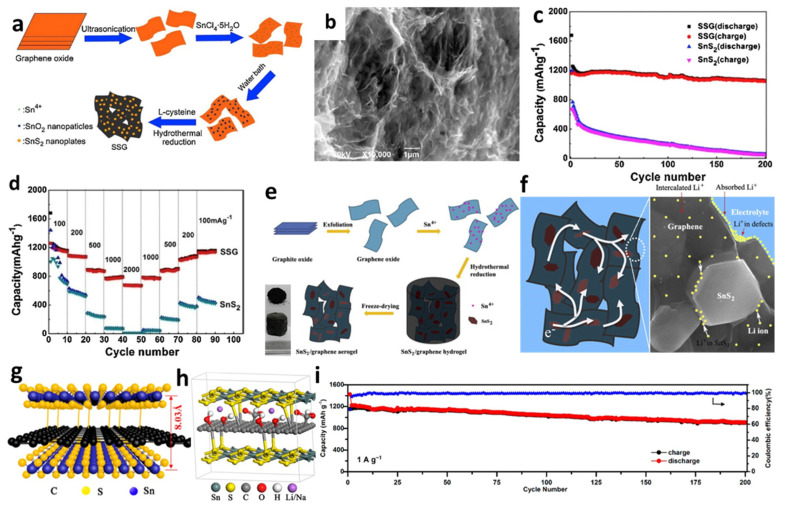
(**a**) Schematic formation process, (**b**) an SEM image, (**c**) cycling performance, and (**d**) rate capability of SSG. Reprinted with permission from Ref. [148]. Copyright 2015 Elsevier. (**e**) Fabrication process, and (**f**) schematic representation of electron transmission and lithium ions storage of SnS_2_/GAs. Reprinted with permission from Ref. [150]. Copyright 2013 Elsevier. (**g**) Schematic illustration, (**h**) molecular model, and (**i**) high rate cycling performance of SnS_2_/RGO/SnS_2_. Reprinted with permission from Ref. [149]. Copyright 2019 ACS.

Meanwhile, due to the high porosity, low density, and large pore volume, several self-assembled graphene aerogels (GAs) and composites of 3D graphene-embedded metal or metal oxide nanopaticles have been successfully manufactured using various approaches [150,151,152,168,169,170]. For instance, Tang et al. [148] prepared a unique 3D SnS_2_/graphene (SSG) composite through transforming SnO_2_ nanoparticles anchored on GO sheets directly into SnS_2_ nanoplates, homogeneously embedded in the graphene frameworks (Figure 9a,b). The diameter of the obtained nanoplates on graphene was about 300 nm. The initial discharge and charge capacities of the cell were 1677 and 1159 mAh/g, respectively. A reversible capacity of 1060 mAh/g was retained after 200 cycles at a current density of 100 mA/g. When the current density declined from 2000 to 100 mA/g, it was found that the reversible capacity could be up to 1100 mAh/g (Figure 9c,d). Jiang et al. [150] successfully fabricated 3D SnS_2_/graphene aerogels (SnS_2_/GAs) via an in situ hydrothermal method for self-assembly of graphene sheets followed by freeze-drying to maintain a stable 3D structure (Figure 9e). Figure 9f illustrates that the cell with SnS_2_/GAs exhibited high-rate capability and cycling stability, which could be ascribed to the unique 3D interconnected architectures of the aerogels and the synergistic effects of the layered SnS_2_ and the graphene, providing enough sites for absorbing Li-ions and shortening transport distance between electrode and electrolyte. Additionally, 3D sandwich-like SnS_2_/graphene/SnS_2_ with expanded interlayer distance was introduced by Jiang et al. [149]. The covalently SnS_2_ nanosheets were decorated on both sides of RGO sheets to form an SnS_2_/RGO/SnS_2_ anode composite. The presence of GO could provide a nucleation site for SnS_2_ and promote SnS_2_ nanoplates aggregate and grow to form fewer layers. SnS_2_ nanosheets were chemically linked to graphene through the C-S bonds to produce a sandwich structure with specific capacities of 844 mAh/g after 200 cycles at a current density of 1 A/g (Figure 9g–i).

**Figure 10:**
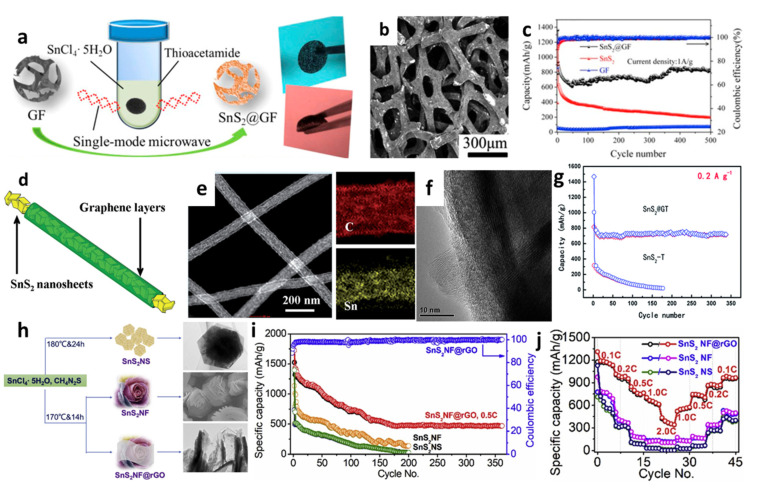
(**a**) Schematic illustration of the formation, (**b**) an SEM image, and (**c**) cycling performance of SnS_2_@GF. Reprinted with permission from Ref. [144]. Copyright 2016 Elsevier. (**d**) Schematic illustration, (**e**) an SEM image and mapping, (**f**) a TEM image, and (**g**) cycling performance of SnS_2_@G. Reprinted with permission from Ref. [158]. Copyright 2014 RSC. (**h**) Schematic diagram and SEM images, (**i**) cycling performance, and (**j**) rate capability of SnS_2_-NF@RGO. Reprinted with permission from Ref. [49]. Copyright 2019 Elsevier.

Apart from nanosheets, Ren et al. [144] introduced SnS_2_ nanoflakes on the 3D graphene foams (GFs) using a single-mode microwave hydrothermal technique (Figure 10a,b). The composite SnS_2_@GF electrode provided a high capacity of 818.4 mAh/g at a high current density of 1.0 A/g after 500 cycles, as seen from Figure 10c. The GF served as a 3D framework for SnS_2_ nanoflakes loading and this conductive porous matrix was convenient for rapid electron transport, reduced the strain during the intercalation/extraction process, and provided a large electrode/electrolyte contact area. A new nanocable-like structured SnS_2_–graphene network was fabricated by Kong et al. [158], in which graphene layers were rolled up to embody SnS_2_ nanosheets with a thickness of around 10 nm. SnS_2_@G nanocable showed the initial discharge and charge capacities of 1334 and 764 mAh/g, respectively (Figure 10d–f). Figure 10g presents that the composite maintained the reversible capacity of 720 mAh/g at 200 mA/g up to 350 cycles with over 93.5% capacity retention. This might be attributed to the unique structure design which released the volume change of SnS_2_ during discharge–charge cycles and promoted easy access of electrolytes to dynamic anode materials. Liu et al. [49] synthesized nanoflower-based SnS_2_@RGO (SnS_2_-NF@RGO) composite anodes for LIBs (Figure 10h). The initial specific capacities of SnS_2_-NS and SnS_2_-NF were 1300 and 1100 mAh/g, respectively, and gradually decreased to below 200 mAh/g after 200 cycles under 615.5 mA/g, while SnS_2_-NF@RGO maintained reversible capacity of 525 mAh/g after 360 cycles and capacities of 1211.8, 1021.7, 809.1, 708.1, 412.5, 509.6, 751.5, 820.3, and 923.5 mAh/g under 123.1, 246.2, 615.5, 1231, 2462, 1231, 615.5, 246.2, and 123.1 mA/g, respectively (Figure 10i,j). It revealed good capacity retention through the layer structure of RGO additives, which gave better conductivity between SnS_2_-NF/electrolyte interfaces and minimized the self-aggregation during the Li^+^ insertion/deinsertion processes.

The electrochemical performance of SnS_2_/graphene anodes is summarized in Table 3. It can be seen that SnS_2_/graphene-based anodes have attracted great attention thanks to the synergistic interaction of SnS_2_ and graphene. On one hand, the graphene sheets could not only prevent the aggregation of microscopic SnS_2_, but also significantly improve the electrode’s electronic conductivity and buffer volume changes during charge/discharge processes. The inclusion of SnS_2_ between graphene sheets, on the other hand, could successfully prevent graphene restacking. In recent years, a variety of methods have been utilized to make SnS_2_-G nanocomposites, each with its own set of benefits. For example, by simply altering the reaction conditions and additives, hydrothermal/solvothermal methods and other solution-based methods are most typically employed to create SnS_2_/graphene nanocomposites with various nanostructures. SnS_2_ nanostructures, such as nanoparticles, nanorods/nanowire, 2D nanosheets/films, and 3D nanoflowers, can reduce volume change during charge/discharge and can shorten the diffusion length of Li-ions, which is critical for boosting cells’ rate capability and cycle stability.

## 5. Summary and Outlook

SnS_2_/carbon composites have been considered as an appealing family of high-capacity anode materials for next-generation LIBs. This review provides a comprehensive overview of the most significant advances in their microstructure, Li storage instrument, combination, and electrochemical characteristics. Specific accentuation has been put on handling the rest of the issues of SnS_2_-based anode materials through a reasonable basic structure (i.e., building remarkable nanostructures, different morphology, and creating SnS_2_/carbon-based composites). Besides, different procedures can be taken for electrochemical execution upgrade, such as securing controlled pre-lithiation and polymer fastener enhancement.

The creators suggested that the micropores could adequately mitigate the volume changes of SnS_2_ nanoparticles and forestall the breakdown of the permeable structure. The high explicit surface zone encourages the effective contact of dynamic materials with electrolytes. Among different carbon materials, compositing SnS_2_ with graphene sheets is one of the hotly debated ongoing exploration issues in recent years since the synergistic impact between SnS_2_ and graphene can strikingly improve the anode’s electrochemical performance. On the one hand, the graphene sheets could not just forestall the agglomeration of small SnS_2_ particles, cradle the volume change during charge–release forms, and essentially upgrade the terminal’s electronic conductivity. The diverse nanostructures of SnS_2_, including nanoparticles, nanorods/nanowires, nanosheets, nanoflowers, and 3D nanospheres, can also ease the volume change during the charge–release process.

In this paper, we focused on the morphological structure of the SnS_2_ material. In the case of pure SnS_2_-based anodes, particle size has a significant impact on discharging capacity. SnS_2_, with smaller particle size, showed better capacity retention and discharging capacity, and also provided more a specific area for volume change expansion. Nanoflower based structures with more active sites for Li-ion insertion significantly improve capacity retention and discharging capability at high current rates. In case of hybrid materials, the specific area and morphology of the other component also play a vital role in capacity performance. As we discussed in the graphene section, sandwich-like nanosheet structures could reduce the Li-ion diffusion distance and have shown excellent CE and rate capability. However, the dispersion length of Li particles is an extremely important factor in improving the reversible limit and cycling dependability. Graphene-based materials are growing rapidly as an incredibly adaptable 2D material for electrochemical energy storage systems, which have aided batteries in achieving excellent high capacities and rate capability due to their optimized interlayer spacing and proprietary chemistry. These accomplishments are a result of graphene’s inherent features, which include strong electrical conductivity, a defined structure, and the capacity to sustain adaptations, allowing for the electrodes to be tailored to a specific application.

**Figure 11:**
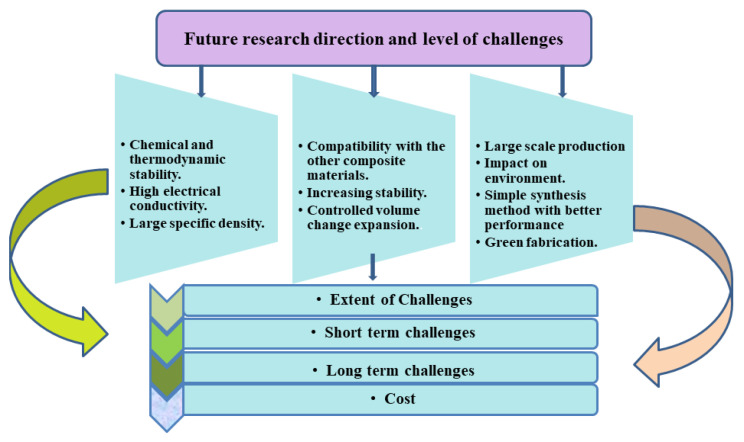
Future research direction and level of challenges.

There is still a long way to go before the use of SnS_2_/graphene composites. Although some of the procedures listed are fairly easy after obtaining graphene or GO, one of the major difficulties is the question of how to further simplify the process of manufacturing graphene. Furthermore, further work is required to alter the mechanical characteristics of the SEI layer, such as SnS_2_/graphene-active materials, binders, electrolytes, and electrolyte additives, in order to achieve improved cycle performance and CE (Figure 11). Meanwhile, high energy consumption in material and battery production, depletion of critical raw material resources, and low degradation rates are incompatible with the current sustainability of LIBs, and could result in a severe environmental impact and uncertain production conditions in the future, which also need to be taken under consideration for future work. However, it is certain that SnS_2_-based anode materials will make tremendous advances in the near future due to the ongoing and unwavering efforts throughout the world, which will play an increasingly essential and active role in next-generation high-energy-density LIBs.

## Figures and Tables

**Table 1:** Electrochemical performance of pure SnS_2_-based anodes with various morphologies.

Materials	Morphology	Size/Thickness (nm)	Initial CE	Specific Capacity (mAh/g)	Rate Performance (mAh/g)	Ref.
SnS_2_	Nanoparticle	N/A	<50%	400/50 mA/g, 25th cycle	N/A	[83]
SnS_2_	Nanoparticle	30	N/A	404/50 mA/g, 30th cycle	N/A	[67]
SnS_2_	Nanosheet	2–26	43%	500/323 mA/g, 50th cycle	~368/3.2 A/g	[50]
SnS_2_ (3D)	Microsphere	10	34%	570/650 mA/g, 100th cycle	264/6.5 A/g	[95]
SnS_2_ (2D)	Nanoplate	10	36%	521/100 mA/g, 50th cycle	340/3 A/g	[96]
SnS_2_ (2D)	Nanoplate	35	73%	935/200 mA/g, 30th cycle	370/5 A/g	[97]
SnS_2_ (2D)	Nanoplate	16	~50%	583/323 mA/g, 30th cycle	N/A	[94]
SnS_2_ (NW)	Nanowall	<50	36%	700/0.3 C, 40th cycle	400/1.2 C	[98]
SnS_2_	Nanosheet	1–3	~44%	900/1 C, 10th cycle	360/5 C	[99]
SnS_2_ (3D)	Nanoflower	5–10	~32%	502/200 mA/g, 50th cycle	N/A	[66]
SnS_2_ (3D)	Nanoflower	30	~30%	519/100 mA/g, 50th cycle	297/0.8 A/g	[101]
SnS_2_ (3D)	Nanoflower	50	N/A	549/100 mA/g, 100th cycle	210/1 A/g	[102]

**Table 2:** Electrochemical performance of carbon-coated SnS_2_ and SnS_2_-CNT-based anodes.

Materials	Morphology	Size/Thickness nm	Initial CE	Specific Capacity (mAh/g)	Rate Performance (mAh/g)	Ref.
C-SnS_2_	Nanoparticle	80	41%	668/50 mA/g, 50th cycle	600/645 mA/g	[121]
SnS_2_/C-x	Nanoparticle	60	80.8%	540/100 mA/g, 100th cycle	300/2 A/g	[122]
MC-SnS_2_ NS	Nanoplate	5–15	N/A	428.8/100 mA/g, 50th cycle	150/1 A/g	[123]
C-SnS_2_	Nanoplate	75	78%	800/500 mA/g, 300th cycle	796/2 A/g	[56]
SnS_2_@MWCNT	Nanosheet	80–100	37.2%	420/100 mA/g, 50th cycle	310/500 mA/g	[126]
SnS_2_@MWCNT	Nanoflake	N/A	37%	510/100 mA/g, 50th cycle	329/500 mA/g	[133]
CPN@SnS_2_	Nanosheet	N/A	89.8%	699.2/60 mA/g, 100th cycle	553.5/1.5 A/g	[127]

**Table 3:** Electrochemical performance of SnS_2_/graphene-based anodes.

Materials	Morphology	Size/Thickness nm	Initial CE	Specific Capacity (mAh/g)	Rate Performance (mAh/g)	Ref.
G/SnS_2_	Nanoparticle	30	29.6%	351/200 mA/g, 50th cycle	N/A	[54]
RGO-SnS_2_	Nanoparticle	100	63.44%	405/0.5 C, 80th cycle	200/5 C	[53]
SnS_x_-G,1 < x < 2	Nanoparticle	5	69%	860/0.2 C, 150th cycle	450/2 C	[137]
SnS_2_/graphene	Nanocrystal	3–5	71.5%	564/0.2 C, 60th cycle	242/5 C	[138]
SnS_2_-graphene	Nanoparticle	5–20	63.2%	903/200 mA/g, 50th cycle	500/1.6 A/g	[143]
SnS_2_/GNS	Nanoparticle	2–3	~69.9%	577/59.1 mA/g, 50th cycle	200/591 mA/g	[147]
SnS_2_/RGO	Nanocrystal	10–40	35%	644/500 mA/g, 50th cycle	430/1 A/g	[154]
SnS_2_/RGO	Nanocrystal	3–4	78.7%	1034/0.1C, 200th cycle	415/5 C	[156]
SnS_2_ NP/GNs	Nanoparticle	4	49%	631.4/100 mA/g, 150th cycle	378/20 A/g	[140]
RGO/SnS_2_/TiO_2_	Nanoparticle	~10	64.3%	485/0.5 A/g, 200th cycle	303/2 A/g	[141]
SnS_2_/graphene	Nanoparticle	3	74.4%	1480/0.2 A/g, 50th cycle	666/10 A/g	[159]
G-SnS_2_	Nanoplate	7	38%	650/50 mA/g, 30th cycle	230/6.4 A/g	[142]
SnS_2_/graphene	Nanoplate	2–5	69%	704/387 mA/g, 100th cycle	303/6.45 A/g	[155]
SnS_2_-G	Nanoplate	~3.6	73%	826/500 mA/g, 200th cycle	498/8 A/g	[151]
SnS_2_/G	Nanoplate	N/A	42.4%	920/100 mA/g, 50th cycle	600/1 A/g	[139]
SnS_2_/GAs	Nanoplate	200	37%	656/50 mA/g, 30th cycle	240/1 A/g	[150]
SnS_2_/graphene	Nanoplate	300	69%	1060/100 mA/g, 200th cycle	670/2 A/g	[148]
SnS_2_/G-CNT	Nanosheet	10–30	63%	1017/100 mA/g, 100th cycle	634.6/2 A/g	[170]
SnS_2_/GNS	Nanosheet	20–25	83.7%	1114/100 mA/g, 30th cycle	870/1 A/g	[145]
L-SnS_2_/G	Nanosheet	5	74.16%	773/200 mA/g, 180th cycle	567/2 A/g	[146]
SnS_2_-graphene	Nanosheet	N/A	~71%	570/0.2 C, 30th cycle	N/A	[153]
SnS_2_/RGO	Nanosheet	10	55.6%	514/1.2 A/g, 300th cycle	447/8 C	[157]
SnS_2_/RGO/SnS_2_	Nanosheet	4.43	81%	1357/100 mA/g, 200th cycle	844/10 A/g	[151]
SnS_2_@GT	Nanorod	10	57.3%	720/0.2 A/g, 350th cycle	247/5 A/g	[158]
SnS_2_@GF	Nanoflakes	N/A	69.6%	818.4/1 A/g, 500th cycle	160.9 /5 A/g	[144]
GNS@MoS_2_@SnS_2_	Nanoflakes	20	66%	743/80 mA/g, 100th cycle	710/320 mA/g	[152]
SnS_2_NF@RGO	Nanoflower	N/A	~78%	525/615.5 mA/g, 360th cycle	412.5/2462 mA/g	[49]

## Data Availability

Not applicable.
